# A Comparison of Two Different Slaughter Systems for Lambs. Effects on Carcass Characteristics, Technological Meat Quality and Sensory Attributes

**DOI:** 10.3390/ani11102935

**Published:** 2021-10-11

**Authors:** Elin Stenberg, Katarina Arvidsson-Segerkvist, Anders H. Karlsson, Aðalheiður Ólafsdóttir, Óli Þór Hilmarsson, María Gudjónsdóttir, Guðjón Thorkelsson

**Affiliations:** 1 Department of Animal Environment and Health, Swedish University of Agricultural Sciences, P.O. Box 234, 53223 Skara, Sweden; katarina.segerkvist@slu.se (K.A.-S.); Anders.H.Karlsson@slu.se (A.H.K.); 2 Matís OHF, Vínlandsleið 12, 113 Reykjavík, Iceland; adalheiduro@matis.is (A.Ó.); oli.th.hilmarsson@matis.is (Ó.Þ.H.); mariagu@hi.is (M.G.); gudjont@hi.is (G.T.); 3 Faculty of Food Science and Nutrition, University of Iceland, Aragata 14, 101 Reykjavík, Iceland

**Keywords:** lamb, pH, meat colour, cooking loss, Warner–Bratzler shear force, selected breeding, slaughter system

## Abstract

**Simple Summary:**

Slaughter systems for lambs can differ in many ways, as they are optimised to meet site-specific conditions, and may affect carcasses differently. It is therefore important to assess whether different slaughter systems affect meat quality parameters such as colour and tenderness, which are important meat quality factors from a consumer perspective when buying a meat product. This study investigated whether two slaughter systems differing in stunning method, electrical stimulation of carcasses and chilling regime resulted in differences in quality attributes in meat from intact male lambs. It also examined whether breeding for carcasses with a higher incidence of lean muscle and less fat content has affected tenderness scores for Icelandic lambs over time. The results showed that the two slaughter systems tested did not affect meat quality parameters to any large extent. Further analysis showed an increase in mechanical tenderness values in meat samples from the Icelandic lamb population over time, which could be due to selective breeding for carcasses with higher muscle and less fat content.

**Abstract:**

Two slaughter systems for lambs and their effects on meat quality in terms of texture, colour and sensory attributes were compared. The slaughter systems differed in methods for controlling *rigor mortis* and carcass chilling. One slaughter system (large-scale) used electrical stimulation and fast chilling of carcasses, while the other system (small-scale) did not use electrical stimulation and applied slower chilling, with carcass temperature decreasing over a longer period after slaughter. Ten pairs of ram lamb twins were selected, and one of each pair was slaughtered at the large-scale abattoir and the other at the small-scale abattoir. Carcass weight, conformation, fatness, pH and temperature were recorded. *Musculus longissimus thoracis et lumborum* was analysed for colour, cooking loss, Warner–Bratzler shear force and sensory attributes. For meat quality attributes, the only differences were found in meat colour L* (lightness; *p* = 0.0073), sensory attribute “appearance colour” (*p* = 0.0089) and “fatty flavour” (*p* = 0.0554). Meat from the small-scale abattoir was darker in colour and had a more fatty flavour than the meat from the large-scale abattoir. For sensory attributes (apart from colour), no significant differences were found between the two abattoir systems.

## 1. Introduction

Lamb production is a traditional and economically important part of Icelandic agriculture. The production system for lambs in Iceland generally involves lambs being born in May and reared on summer pasture until gathering in August, when lamb slaughter starts. The slaughter season usually ends in November. Lamb has high domestic food value and it is of critical importance to produce high-quality meat that satisfies consumer demands. An investigation in 2003 on the tenderness of Icelandic lamb found low shear force values (mean 1.8 kg/cm^2^), indicating tender meat [1]. However, a more recent study indicated a decrease in tenderness of *Musculus longissimus dorsi* meat from Icelandic lamb during the past two decades [2]. Probable reasons for this trend are (i) ongoing breeding for increased muscle and decreased fat using progeny testing, based on ultrasound measurements of live animals for animal selection [3], and (ii) changes in slaughter practices, with faster chilling of lamb carcasses and low-voltage electrical stimulation. It is known that rapid chilling of carcasses can affect meat tenderness negatively, by increasing the risk of cold shortening [4]. Electrical stimulation of carcasses can prevent cold shortening when applied in combination with fast chilling of the carcass after slaughter [5]. To prevent cold shortening through accelerating pH decline, electrical stimulation within 30 min post-mortem can be applied [6]. Electrical stimulation can therefore be used to improve the eating quality of meat by controlling the pH decline in relation to muscle temperature [7]. However, growing interest in local produce and in procuring meat directly from farms is resulting in small local abattoirs using traditional slaughter methods being set up.

It is important to understand how the slaughter system can affect meat quality parameters, in order to optimise it and avoid negative effects on quality. It is also important to determine how breeding has affected meat quality attributes such as tenderness, in order to adapt or make changes in the breeding goals if necessary. Hence selection for lean meat yield could negatively affect meat quality as perceived by consumers [8]. The aims of this study were therefore to investigate whether small-scale slaughter systems affect quality attributes in lamb meat compared with large-scale slaughter systems and to assess whether breeding for carcasses with a higher incidence of muscle and less fat has affected lamb tenderness scores over time.

## 2. Materials and Methods

Ten pairs of intact ram lamb twins of the breed Icelandic sheep were included in the study. One of each pair was slaughtered in a large-scale abattoir slaughtering 2500 lambs/day and the other in a small-scale abattoir slaughtering 75 lambs/day. All lambs were raised on the same farm in south-east Iceland. The lambs were born in May and grazed with their mothers for the first weeks on cultivated pasture and then on natural pasture (aftergrass) until mid-September. Before slaughter, all lambs were kept in lairage for 10–12 h overnight at the abattoir without feed, but with free access to water. Lambs were on average 160 days at slaughter. All carcasses were visually assessed by trained personnel according to the EUROP scale for conformation (classes 1–5, with class 5 being the most swelling) and fatness (classes 1–6, with class 6 being the fattiest). All carcasses were hung by the Achilles tendon after dressing (removal of skin, legs, head and visceral organs).

### 2.1. Large-Scale Abattoir

Transport from the farm to the large-scale abattoir involved three hours in a ventilated lorry. The lambs were stunned electrically (head to back) with 110 V, 50 Hz for 5 s and slaughtered by exsanguination. After dressing and evisceration, the carcasses were electrically stimulated (10 A, 80 V for 60 s) before entering the chiller. The carcasses were then chilled at 2–4 °C for 30 h before sampling of *M. longissimus thoracis et lumborum* (LTL).

### 2.2. Small-Scale Abattoir

Transport time from the farm to the small-scale abattoir was 30 min. All lambs in that system were stunned with a captive bolt pistol and slaughtered by exsanguination. The carcasses were kept at 10–15 °C for the first six hours after slaughter and then chilled at 3–4 °C for 30 h until sampling of the LTL muscle.

### 2.3. Carcass Weight Loss during Chilling

The carcasses were weighed upon arrival at the chiller (hot carcass weight) and after chilling (cold carcass weight). The difference in weight was used for calculating carcass weight loss (%) as:

% Carcass weight loss = (Hot carcass weight (g) – Cold carcass weight (g))/Hot carcass weight × 100.

### 2.4. Sampling, Packaging and Handling of the LTL Muscle

The LTL muscle with subcutaneous fat was cut from the left side of all carcasses, between the last lumbar vertebrae and the seventh thoracic vertebrae, 24–30 h after slaughter. The whole muscle was labelled, weighed and vacuum-packed in 25 cm × 35 cm bags, and then aged for 6–7 days at 2–4 °C. All LTL samples were analysed fresh for both technological and sensory quality attributes. Unfortunately, two samples from the large-scale abattoir were frozen prior to analysis, and are thus not included in the results for colour, cooking loss, Warner–Bratzler shear force (WBSF) and sensory attributes. 

### 2.5. pH Measurements

The pH and temperature value in all carcasses were measured 24 h after slaughter, by inserting a probe (Seven2Go pro, Metler Toledo, Schwerzenbach, Switzerland) into the LTL muscle between the 12th and 13th ribs. 

### 2.6. Colour

Colour was measured before vacuum packaging on fresh, uncooked LTL one day after slaughter, using a Minolta CR-300 colorimeter (Konika Minolta, Tokyo, Japan) with a D65 light source. The instrument provided information about the lightness (L* value), redness (a* value) and yellowness (b* value) of the muscle samples. The LTL muscle was allowed to bloom for one hour before measuring. Each attribute was measured in triplicate for each muscle sample.

### 2.7. Warner–Bratzler Shear Force (WBSF)

Instrumental LTL tenderness, expressed as shear force, was analysed on cooked LTL, using a Warner–Bratzler knife (TA-7), with a guillotine block, connected to a texture analyser (TA.HD Plus Connect, Godalming, Surrey, UK), and a head speed of 2.5 mm/s. Samples with dimensions 1.0 cm × 1.0 cm × 3.0 cm (width × height × length), cut orthogonal to the fibre direction, were used for these measurements.

### 2.8. Sensory Evaluation

The sensory evaluation was carried out using a trained panel (Generic descriptive analysis) of a total of 11 panellists, with 6–10 panellists at each of five occasions depending on availability [9]. The software used for checking panel performance was PanelCheck V1.4.0 (Nofima, Tromsö, Norway) and that used for data collection was FIZZ (Version 2.50B, Biosystémes, Couternon, France). PanelCheck is a tool that uses several plots to evaluate results from descriptive analyses, which helps the user to identify the performance of individual assessors. The meat was evaluated in five sensory sessions, with four different samples per panellist tested in each. Each panellist tested one sample per LTL from the same location within the muscle. Hence, all LTL was divided into ten samples to provide each panellist with a sample of LTL from the same muscle location in each session. All samples were coded with random three digit numbers and presented to the panellists in a random order. Latin square order was used to obtain randomized order of samples. The meat was cooked sous vide (Anova Precision Cooker, Anova Culinary Inc., San Francisco, CA, USA) at 68 °C for one hour and then flash-fried in a dry pan at a high temperature for 30 s on each side. The cooked meat was cut into 2 cm thick slices, placed in aluminium containers and served to the panel while still warm. The panellists were placed in separate cubicles with standardised light. They were offered crackers and water to avoid residual flavours between samples. Panellists evaluated each sample on a scale from 1 to 100 for odour (frying, sour, fatty and liver), appearance (colour), flavour (frying, sour, fatty, sweet and liver) and texture (softness, tenderness, juiciness and mushiness). 

### 2.9. Cooking Loss

All LTL samples were weighed before and after cooking, to calculate cooking loss (%).

### 2.10. Statistics

Statistical analysis was performed using Proc Mixed of the Statistical Analysis Software (SAS) [10], with slaughter system as a fixed effect and twin pair of lambs as random effect, using model 1 below. A general Satterthwaite approximation for the denominator degrees of freedom was performed, using SATTERTH option in SAS. Differences were considered significant at *p* ≤ 0.05 and a tendency for significance was assumed at 0.05 < *p* ≤ 0.10. Carcass characteristics and technological meat quality attributes are presented as mean and standard deviation, respectively.

Model 1: Y_ij_ = µ + S_i_ + P_j_ + e_ijk_ (1)

where Y_ij_ is the dependent variable, µ is the grand mean, S_i_ is the fixed effect of slaughter system, P_j_ is the random effect of twin pair, and e_ijk_ is the residual error.

## 3. Results

### 3.1. Carcass Characteristics and Technological Quality Attributes

There was a difference in fatness scoring (*p* = 0.0002) between the abattoirs, with the small-scale abattoir obtaining a higher score (3.5) than the large-scale abattoir (2.3) (Table 1). There was also a difference (*p* = 0.0073) in colour (L*) between the abattoirs, with the large-scale abattoir having a higher L* value than the small-scale abattoir (Table 1). A tendency for significance was found in temperature_24_ where the small-scale abattoir had a bit lower temperature (4.3 °C) compared to the large-scale abattoir (5.0 °C). There were no significant differences between the slaughter systems for carcass conformation, hot carcass weight, cold carcass weight, carcass weight loss, pH_24h_, cooking loss or WBSF (Table 1). Individual data for each animal regarding carcass characteristics and technological quality attributes can be found in Tables S1 and S2.

### 3.2. Sensory Attributes

In line with the technological colour measurements, a difference (*p* = 0.0089) was found for the sensory parameter “colour appearance”, with lamb from the small-scale abattoir obtaining a higher score (33, i.e., darker) than lamb from the large-scale abattoir (30) (Table 2). A difference (*p* = 0.0370) was also found for the parameter “fatty flavour”, for which lamb from the small-scale abattoir had a higher score (18) than lamb from the large-scale abattoir (15) (Table 2). No differences were found for the different odour and texture attributes, or for frying, sour, sweet and livery flavour (Table 2). Individual data for each animal regarding sensory attributes can be found in Table S2.

## 4. Discussion

The two slaughter systems compared in this study differed in multiple ways, such as type of stunning, use of electrical carcass stimulation and chilling regime. Despite these major slaughter and carcass handling differences, only a few differences were found in technological and sensory meat quality attributes. This indicates that both systems investigated can be used in practice without compromising these meat quality attributes under the circumstances stated. This was an unexpected finding, as previous studies have shown that numerous factors during pre-slaughter, slaughter and post-slaughter can all contribute to variation in meat quality attributes, as reviewed by Sañudo et al. [11]. The results from the present study indicate good robustness for both slaughter systems tested and the different treatments within each system. A limitation of this study could thus be that all animals came from the same farm and got slaughtered on the same day. However, in this way, we could not only limit the genetic effects by using twin lambs, and we also reduced the effect of variation between farms as well as day of slaughter which let us focus on the effect of slaughter system.

The stunning method used in the abattoirs was either captive bolt or electrical stunning. Previous studies have found that the stunning method does not affect meat quality attributes [12]. It has also been shown that the use of electrical stunning head-to-leg, combined with low voltage electrical stimulation of the carcass, can increase the rate of pH decline post-slaughter, compared with electrical head-only and captive bolt stunning with low-voltage electrical stimulation. While not measured in this experiment, electrical stunning increasing pH decline could be interpreted as a positive effect prior to fast chilling of carcasses shortly after stunning, to avoid cold shortening [13]. Therefore it can be concluded that the electrical stunning combined with the fast chilling approach used in the large-scale abattoir may be beneficial to avoid cold shortening.

The pairs of twin lambs included in this study were almost identical in carcass weight and conformation score, but there was a significant difference in fat score between the abattoirs. This difference probably arose because the classification was performed by different individuals in the two abattoirs [14], since a review by Craigie et al. [15] have shown that carcass scoring is not consistent between individual classifiers.

Both pH_24_ and cooking loss were unaffected by slaughter system. Both slaughter systems resulted in a pH_24_ value that met recommendations by Meat Standards Australia (MSA), with an upper limit of 5.7 [16]. Hence, based on recommendations from the MSA the pH_24_ values recorded in the present study would have positive effects on eating quality attributes such as tenderness. Cooking loss was unaffected by electrical stimulation (large-scale abattoir only), supporting previous findings [5,6,17]. It can therefore be concluded that the slaughter systems had similar effects on pH_24_ and cooking loss in the lamb samples.

Meat colour is a complex meat quality factor that can be affected by various factors linked to the production system, the slaughter system and the animal material [18]. The results obtained here for a* and b* were similar for both slaughter systems, supporting previous findings [6,14,19]. Hopkins and Ferrier [6] showed that, despite a more rapid pH decline in stimulated carcasses, there was no effect on meat colour. Even with similar pH_24_ values in lamb samples, a difference in L* was found between the slaughter systems in the present study, which is in line with findings by Pouliot et al. [5]. A darker colour was observed for meat samples from the small-scale abattoir compared with the large-scale abattoir. Differences in lightness have previously been explained by different myoglobin concentrations in muscle due to differences in animal age [20], or to changes during the first hour of meat blooming due to different temperatures in muscle at rigor development [21]. The reason for the difference in lightness in the present study is unclear since there was no difference in animal age. However, this difference was also detected on cooked meat by the sensory panel, which recorded a difference in the attribute “colour appearance” between the two abattoirs, with meat from the small-scale treatment being rated as darker than meat from the large-scale abattoir. At the point of purchase, based on previous findings, consumers would consider meat from both slaughter systems studied here to be of acceptable lightness [22]. Therefore it can be assumed that, while the difference in lightness might be visible to the consumer, it would probably not influence the acceptability of lamb from either of the slaughter systems compared here. 

Another difference between the abattoirs was the use of electrical carcass stimulation, as low-voltage electrical stimulation of the carcasses was used only at the large-scale abattoir. Previous studies have compared different electrical settings (V, Hz, A) and different durations of electrical stimulation (s), with non-consistent results. Some studies have found differences in WBSF when using low-voltage electrical carcass stimulation compared with a control group [5,17,23,24,25,26,27], while other studies have found no differences [6,19,28]. A study by Polidori et al. [26] concluded that low-voltage electrical stimulation of lamb carcasses may reduce potentially negative risks to meat quality associated with fast chilling of carcasses immediately after dressing. This may be because low-voltage electrical stimulation induces glycolysis [29]. However, Pommier et al. [28] used low-voltage stimulation in their study and, based on their results, they suggest that high-voltage electrical stimulation is required to get a rapid pH decline that allows early freezing of meat without negative consequences for meat quality attributes. It is debatable whether high-voltage electrical stimulation is required to allow fast chilling in order to avoid cold shortening. The results from the present study suggest that low-voltage stimulation is sufficient to allow fast chilling of carcasses without affecting WBSF negatively, at least compared with the small-scale slaughter system without electrical stimulation and a slower chilling process.

When considering WBSF from the perspective of the values themselves, there were no significant differences between the slaughter systems (Table 1). The breeding goals for Icelandic sheep (the only bread present in Iceland) in recent decades have focused on carcass composition traits, such as improved slaughter weight, more muscle and less fat [3]. This selection process has resulted in a change in carcass composition in Icelandic lambs, according to Eiríksson and Sigurdsson [3]. Based on the WBSF threshold of ≤5 kg (~49 N) set by Shorthose et al. [30], the meat from slaughter systems can be interpreted as acceptably tender. However, meat samples from both the large-scale and small-scale systems (WBSF = 49 N and 43 N, respectively), were near or at the threshold. In studies in 2003 of meat from male Icelandic lambs [1,31], lambs just over 4 months of age had WBSF values of 17.2 N (1.75 kg) [1] and 18.3 N (1.87 kg) [31] and 7-month-old lambs had a value of 27.6 N (2.81 kg) [1]. A study in 2018 reported WBSF values for Icelandic lamb of on average 48.2N (4.92 kg) [2], indicating an increase in WBSF due to selective breeding over recent decades. This statement is supported by previous findings that selection for lean meat yield can reduce the consumer eating quality of lamb [8]. In addition, others have found decreasing sensory scores for the traits tenderness, juiciness, flavour and overall liking as an effect of breeding [14]. 

Except for “colour appearance” and “fatty flavour”, the attributes tested in the present study did not differ between the two slaughter systems. Previous studies have found differences in the sensory attributes firmness (less firm with electrical stimulation) [5], flavour (ovine flavour less intense with electrical stimulation) [5], palatability (more palatable with electrical stimulation) [25] and tenderness (more tender with electrical stimulation) [23,24,25] when comparing electrical stimulation to untreated carcasses. However, other studies have found no differences in tenderness [28], flavour [23,25,28] or juiciness [5,23,25,28]. Spanier et al. [32] detected a difference in flavour development in beef during post-mortem ageing, which could explain the tendency for the small-scale abattoir in the present study to have a higher score for fatty flavour. Another explanation for differences in flavour between electrical stimulation and control groups is suggested to be connected to decreased firmness of the meat following electrical stimulation [5]. The meat then requires less chewing and flavour compounds are less likely to be liberated, so the flavour perception is lower [5]. However, there was no significant difference in WBSF or perceived tenderness scores between the system with and without electrical stimulation in the present study, contradicting this suggestion.

## 5. Conclusions

The most important finding from this study was that the two slaughter systems compared, one with electrical stimulation and fast chilling of carcasses and one with electrical stimulation and slower chilling of carcasses, did not result in any differences in meat quality parameters. However, more research is needed to see if the results of this study persist with different animal material and production systems.

## Figures and Tables

**Table 1:** Carcass characteristics, pH_24h_, temperature_24_, colour measurements and cooking loss of lamb slaughtered in the small-scale system (*n* = 10) and large-scale system (*n* = 10/*n* = 8 ^1^; means ± stdev).

Parameters	Small-Scale	Large-Scale	SEM ^2^	*p*-Value ^3^
*Carcass characteristics*				
Hot carcass weight (kg)	19.8 ± 1.2	19.8 ± 1.7	0.46	0.9580
Cold carcass weight (kg)	19.4 ± 1.1	19.3 ± 1.7	0.46	0.9039
Carcass weight loss (%) ^4^	2.44 ± 0.6	2.74 ± 0.2	0.13	0.1476
Conformation score ^5^	3.9 ± 0.3	4.0 ± 0.5	0.13	0.5911
Fatness score ^6^	3.5 ^a^ ± 0.5	2.3 ^b^ ± 0.5	0.16	0.0002
pH_24_^7^	5.65 ± 0.1	5.63 ± 0.1	0.04	0.7118
Temperature_24_ (°C) ^8^	4.3 ± 0.6	5.0 ± 1.0	0.25	0.0986
*Meat quality attributes*				
L*	36.5 ^a^ ± 1.5	38.3 ^b^ ± 1.4	0.47	0.0073
a*	18.8 ± 2.2	20.2 ± 1.0	0.51	0.4928
b*	4.2 ± 1.4	4.9 ± 0.7	0.44	0.5500
Cooking loss (%)	14 ± 3.4	16 ± 2.4	1.01	0.1231
WBSF (N) ^9^	49 ± 15.3	43 ± 9.4	4.22	0.9651

^1^ Regarding large-scale abattoir *n* = 10 for carcass characteristics; *n* = 8 for meat quality attributes. ^2^ Standard error of the mean. ^3^ Differences considered significant at *p* < 0.05 and tending towards significance at 0.05 < *p* ≤ 0.10. ^a,b^ = Mean values within rows with different superscripts differ significantly. ^4^ Difference between hot and cold carcass weight (%). ^5^ Scoring into five classes, with five being the highest (very good conformation) and one being the lowest (very poor conformation). ^6^ Scoring into six classes, with six being the highest (very high fat) and one being the lowest (very low fat). ^7^ pH at 24 h after slaughter. ^8^ Temperature at 24 h after slaughter. ^9^ Warner–Bratzler shear force measured in Newton.

**Table 2:** Warner–Bratzler shear force values and sensory analyses comparing lamb slaughtered in the small-scale system (*n* = 10) and large-scale system (*n* = 8).

Parameters		Small-Scale	Large-Scale	SEM ^1^	*p*-Value ^2^
Odour attributes ^3^	Frying	33	33	2.58	0.5446
	Sour	13	12	1.08	0.1398
	Fatty	28	27	1.90	0.9660
	Liver	29	30	1.53	0.5053
Appearance attribute ^3^	Colour	33 ^a^	30 ^b^	1.85	0.0089
Flavour attributes ^3^	Frying	26	23	1.93	0.8533
	Sour	24	26	2.23	0.1054
	Fatty	18	15	1.36	0.0370
	Sweet	9	9	0.57	0.5045
	Liver	41	41	1.80	0.7850
Texture attributes ^3^	Soft	47	53	4.67	0.8389
	Tender	46	50	5.24	0.8131
	Juicy	49	50	3.57	0.7513
	Mushy	14	17	1.39	0.3099

^1^ Standard error of the mean. ^2^ Differences considered significant at *p* ≤ 0.05. ^a,b^ = Mean values within rows with different superscripts differ significantly. ^3^ Sensory attributes were scored on a scale from 0–100, with 100 being the highest score.

## Data Availability

Not applicable.

## Supplementary Materials

The following are available online at https://www.mdpi.com/article/10.3390/ani11102935/s1, Table S1: Individual data on pH and carcass characteristics (Small-scale = Small-scale slaughter system, Large-scale = Large-scale slaughter system). Table S2: Individual data on technological meat quality attributes (colour, cooking loss and WBSF) and sensory attributes (Small = Small-scale slaughter system, Large = Large-scale slaughter system).
