# Supplementary material for: A Comparison of Two Different Slaughter Systems for Lambs. Effects on Carcass Characteristics, Technological Meat Quality and Sensory Attributes
Source: Animals (Basel). 2021 Oct 11;11(10):2935. doi: 10.3390/ani11102935 (PMC8532808; doi:10.3390/ani11102935)
Supplement: Supplementary file 1 [file animals-11-02935-s001.zip › animals-1387888-supplementary.pdf]

Supplementary data

Table S1. Individual data on pH, temperature and carcass characteristics (Small-scale = Small-scale slaughter system, Large-scale = Large-scale slaughter system, CW = carcass weight).

Slaughter system	Animal ID	Lamb pair	pH_{24}[a]	$Temp_{24}$[a]	Conf. Score[b]	Fatness score[c]	Hot CW (kg)	Cold CW (kg)	CW loss (%)[d]
Small-scale	1189	1	5.66	4.1	4	3	20.8	20.3	2.2
Small-scale	1581	2	5.51	5.5	4	4	18.0	17.7	1.9
Small-scale	1685	3	5.62	5.2	4	4	19.8	19.3	2.3
Small-scale	1465	4	5.59	5.4	3	4	20.3	19.9	2.0
Small-scale	1533	5	5.63	5.4	4	4	20.3	19.5	3.9
Small-scale	1812	6	5.93	5.6	4	3	21.0	20.5	2.4
Small-scale	1570	7	5.68	4.1	4	3	17.8	17.4	2.2
Small-scale	1389	8	5.51	4.8	4	3	20.3	19.8	2.5
Small-scale	1699	9	5.8	4.8	4	3	19.1	18.6	2.4
Small-scale	1872	10	5.6	4.6	4	4	21.0	20.5	2.6
Large-Scale	2812	6	5.56	4.2	4	2	20.5	19.9	3.0
Large-Scale	2699	9	5.75	5.8	4	2	17.5	17.0	3.0
Large-Scale	2872	10	5.66	4.0	4	3	18.3	17.8	2.5
Large-Scale	2685	3	5.71	5.4	4	2	18.7	18.1	3.1
Large-Scale	2389	8	5.52	3.0	5	3	22.6	22.0	2.6
Large-Scale	2533	5	5.91	2.8	3	2	17.5	17.1	2.6
Large-Scale	2189	1	5.59	4.3	4	2	20.1	19.5	2.8
Large-Scale	2465	4	5.5	4.7	4	2	20.2	19.7	2.5
Large-Scale	2570	7	5.59	5.0	4	2	21.2	20.7	2.6
Large-Scale	2581	2	5.54	3.8	4	3	21.4	20.9	2.7

[a]pH and temperature at 24 hours after slaughter.
[b]Conformation scoring into five classes, with five being the highest (most swelling muscles).
[c]Scoring into six classes, with six being the highest (fattiest).
[d]Difference between hot and cold carcass weight (%).

Table S2. Individual data on technological meat quality attributes (colour, cooking loss and WBSF) and sensory attributes (Small = Small-scale slaughter system, Large = Large-scale slaughter system).

Slaughter system	Small	Small	Small	Small	Small	Small	Small	Small	Small	Small
Animal ID	1189	1581	1685	1465	1533	1812	1570	1389	1699	1872
Lamb pair	1	2	3	4	5	6	7	8	9	10
L*	37.0	38.1	36.2	35.2	34.0	36.3	37.2	35.4	39.3	36.7
a*	19.7	18.8	20.8	20.4	18.4	15.1	17.4	20.8	15.6	21.1
b*	5.3	3.9	5.1	3.6	2.8	2.3	3.2	6.2	3.3	5.9
Cooking loss (%)	14.2	17.4	9.3	16.7	11.9	9.3	19.8	14.4	12.0	13.7
WBSF (N)[a]	87.2	39.6	49.7	38.4	54.0	46.9	56.6	43.4	41.8	32.8
Frying odour[b]	25	30	43	35	29	33	30	46	29	30
Sour odour[b]	9	17	17	9	22	13	11	11	12	8
Fatty odour[b]	28	28	23	31	30	29	22	28	27	29
Liver odour[b]	31	29	30	32	33	25	23	29	24	38
Colour apperance[b]	30	30	42	26	35	31	34	32	34	38
Frying flavour[b]	24	25	26	21	27	23	24	38	27	22
Sour flavour[b]	17	23	25	31	25	28	28	16	26	25
Fatty flavour[b]	17	17	16	14	22	13	18	22	23	17
Sweet flavour[b]	8	9	11	9	7	10	8	10	8	9
Liver flavour[b]	42	47	50	45	37	36	34	38	35	42
Soft texture[b]	40	54	66	28	35	45	36	66	34	69
Tender texture[b]	36	51	63	26	29	53	29	69	29	71
Juicy texture[b]	49	37	60	39	34	54	42	67	42	62
Muschy texture[b]	12	14	15	10	13	19	11	16	12	22
Slaughter system	Large	Large	Large	Large	Large	Large	Large	Large	Large	Large
Animal ID	2812	2699	2872	2685	2389	2533	2189	2465	2570	2581
Lamb pair	6	9	10	3	8	5	1	4	7	2
L*	37.0	40.1	.	39.1	36.4	.	37.5	39.9	38.8	39.0
a*	20.9	19.5	.	20.4	21.3	.	20.2	18.1	19.5	20.7
b*	4.1	5.3	.	5.9	5.2	.	3.9	4.6	4.3	5.5
Cooking loss (%)	18.2	18.5	.	11.8	17.4	.	14.7	15.1	13.3	16.1
WBSF (N)[a]	32.6	37.4	.	49.5	48.3	.	50.7	49.5	43.8	25.5
Frying odour[b]	22	55	.	24	35	.	30	40	36	21
Sour odour[b]	15	10	.	9	17	.	9	12	13	11
Fatty odour[b]	28	25	.	28	31	.	20	32	29	26
Liver odour[b]	35	32	.	29	20	.	27	34	33	30
Colour apperance[b]	27	30	.	34	25	.	27	36	29	25
Frying flavour[b]	20	31	.	22	23	.	20	25	24	19
Sour flavour[b]	30	32	.	19	22	.	19	31	27	32
Fatty flavour[b]	15	18	.	11	15	.	11	14	19	17
Sweet flavour[b]	11	13	.	7	9	.	7	9	8	10
Liver flavour[b]	47	47	.	35	40	.	35	46	44	39
Soft texture[b]	64	64	.	38	47	.	46	48	44	72
Tender texture[b]	58	69	.	34	43	.	45	42	43	72
Juicy texture[b]	57	51	.	38	47	.	50	49	46	62
Muschy texture[b]	19	24	.	11	13	.	17	13	16	26

[a]Warner-Bratzler shear force measured in Newton.

[b]Sensory attributes was scored on a scale from 0-100, with 100 being the highest score.
